# Calcium as an innovative and effective catalyst for the synthesis of graphene-like materials from cellulose

**DOI:** 10.1038/s41598-022-25943-3

**Published:** 2022-12-13

**Authors:** Théotime Béguerie, Elsa Weiss-Hortala, Ange Nzihou

**Affiliations:** 1 Université de Toulouse, Mines Albi, CNRS, Centre RAPSODEE, Campus Jarlard, Route de Teillet, 81013 Albi Cedex 09, France; 2 School of Engineering and Applied Science, Princeton University, Princeton, NJ 08544 USA; 3 Andlinger Center for Energy and the Environment, Princeton University, Princeton, NJ 08544 USA

**Keywords:** Synthesis of graphene, Materials science, Graphene

## Abstract

Pyrolysis of lignocellulosic biomass (hard carbon) produces poorly graphitic biochar. In this study, nano-structured biochars were produced from microcrystalline cellulose using calcium as a non-conventional catalyst. Calcium is abundant, environmental-friendly and widely accessible. Graphitization of calcium-impregnated cellulose was carried out at 1800 °C, a temperature below 2000 °C where the graphitization usually occurs. XRD, Raman spectroscopy, high-resolution TEM together with the in-house numerical tool developed enable the quantification of the graphene fringes in the biochars. The non-impregnated cellulose biochar was composed of short and poorly stacked graphene fringes. The impregnation with 2 wt.% of calcium led to the conversion of the initial structure into a well-organized and less defective graphene-like one. The graphene-like structures obtained were composed of tens of stacked graphene fringes with a crystallite size up to 20 nm and an average interlayer spacing equal to 0.345 nm, close to the reference value of standard hexagonal graphite (0.3354 nm). The increase of the calcium concentration did not significantly improve the crystallite sizes of the graphene-like materials but rather drastically improved their rate. Our results propose a mechanism and provide new insights on the synthesis of graphene-like materials from bio-feedstocks using calcium where the literature is focused on transition metals such as iron and nickel among others. The decrease of the graphitization temperature below 2000 °C should lower the production cost as well as the environmental impact of the thermal graphene-like materials synthesis using biomass. This finding should stimulate further research in the field and broaden the application perspectives.

## Introduction

Graphene is a bidimensional carbon material with one atomic layer as thickness^1^. Graphene sheets are precursors to carbon nanotubes, fullerene, carbon fibers, carbon black or graphite^2–4^. Depending on the characteristics such as the length and the orientation of the graphene sheets, various properties could be developed among which, the electrical conductivity, the mechanical or thermal resistance^5,6^. Therefore, graphene is considered as a high-performance material, promising for a wide range of applications such as batteries, energy storage, electronics, and biology^4,7–11^.

Nowadays, graphene can be produced from fossil feedstocks by either top-down or bottom-up processes^3,12–16^. Top-down processes include mechanical or chemical exfoliation of extracted graphite to isolate the graphene sheets. Bottom-up processes involve the synthesis of graphene from gaseous hydrocarbon precursors. The advantages and drawbacks of each process have been previously discussed in the literature^17,18^. Almost all processes use fossil carbon sources and are high energy demanding. Managing industry-scale and more eco-friendly production of homogenous and defect-free graphene is one of the main challenges to envisage further commercial use of graphene. The production of graphene-like materials from bioresources (biomass, biowastes…) would open ways for new materials with outstanding properties. This approach, as opposed to the standard graphene manufacturing techniques using chemicals and various solvents, would lower the environmental impact of graphene-like materials synthesis.

Cellulose is a renewable resource and the most abundant organic polymer on Earth. Cellulose is used all over the world for paper production and is widely accessible at cheap cost. Its structure is also relatively homogenous among lignocellulosic biomass, compared to lignin or hemicellulose. The main approach for graphene-like materials synthesis from biomass is pyrolysis to generate graphenic-like biochars. Cellulose is composed of a long chain of D-glucose molecules, with exclusively sp^3^ carbons. During cellulose pyrolysis, sp^3^ carbons follow multiple and complex chemical rearrangement to be converted into sp^2^ carbons and form aromatics rings^19^. Because of this, cellulose is not a favorable material for graphitization.

The graphitization of biomass has been previously investigated in the literature. Graphenic materials were previously obtained from a wide range of biomass, such as woody biomass^20–23^, agricultural^24–35^ or food wastes^36,37^, insects^36,38,39^ or sewage sludges^37,40^. These studies showed a poor organization of the graphenic materials even with high temperatures treatments (> 2000 °C).

The use of high temperatures implies a significant energetic cost which limits the industrial development of graphene derived from biomass.

Catalytic graphitization was extensively investigated in the past for the production of graphene-like materials from carbon black^41^. A wide range of mineral species was investigated. Transition metals, especially iron and nickel, were considered as the most promising catalysts for their lower cost and great catalytic power^42–47^.

However, only few research addressed the catalytic graphitization of biomass. Calcium is the fifth most widespread minerals in the world and is inherently abundant in lignocellulosic biomass^48–50^. Calcium is cheap, non-toxic and more environmental-friendly than standard catalysts for graphitization. Alkali (Na, K) and alkaline earth metal (Ca, Mg) are known to be promoters for the pyrogasification of biomass while producing the biochar^51–53^. These metals and transition metals like iron and nickel have demonstrated their impact of the structuration of the carbon structure in biochar. Calcium was previously reported to catalyze the graphitization of charcoal^54,55^. The metal catalyzed graphitization mechanism has been explained in the literature^56–58^. The work mostly referred to iron and to nickel to a lesser extent. It is suggested that both particle size and degree of reduction of the iron catalyst positively impact biochar graphitization. In particular, facets of metal catalyst in reduced form provide regions for and promote the precipitation of graphitic carbon. The metal reduces the barrier for carbon nucleation and makes it easier for graphene sheets to form. High dispersion of calcium in cellulose could be expected thanks to linkage of calcium ions with the carboxyl groups^59^. As such, calcium could possibly be a promising catalyst for the graphitization of cellulose biochar and, by extension, of lignocellulosic biomass.

This study focuses on the effect of Ca-loaded for the efficient graphitization of the resulting biochar. For this purpose, commercial cellulose was impregnated with calcium nitrate prior to pyrolysis at 1800 °C. For clarity reasons, the terms *structure*, *texture* and *nanotexture* will be used as defined by Monthioux et al.^60^. The carbon structure is amorphous, turbostratic or crystalline; the texture defines the organization of the graphene sheets (concentric, aligned…) while the nanotexture describes the length and the stacking of the graphene sheets in the crystal coherence domains.

The carbon organization in the resulting biochar was characterized at a macroscopic (with X-Ray Diffraction, XRD), local (with Raman spectroscopy) and nanoscopic scales (with High-Resolution Transmission Electron Microscopy, HRTEM). XRD provides a general description of the various carbon structures (amorphous, turbostratic, graphene-like) in the samples and estimations of the crystallite sizes. Raman spectroscopy informs about the level of amorphousness and defectiveness of the graphenic structures at the surface of a char particle. HRTEM complements XRD and Raman spectroscopy by investigating at nanoscale the graphenic structures. The results from each characterization technique were further exploited to extract quantitative information about the graphenic structures. Especially, the HRTEM images were processed through a home-made image analysis numerical tool to estimate the crystallite sizes. The combination of experimental methods such as XRD, Raman spectroscopy and HRTEM together with the numerical processing of the data have enable to provide new insights on the mechanism driving the calcium-catalyzed cellulose graphitization.

## Materials and methods

### Biochars origin and preparation

Biochars, named Ca-1 to Ca-8, were produced by pyrolysis of calcium-impregnated microcrystalline cellulose (Sigma Aldrich CAS: 9004-34-6) under N_2_ at 1800 °C. The impregnation was conducted by immersion, for 6 h, of 40 g of cellulose in 200 mL of deionized water with dissolved calcium nitrate under agitation. The impregnated cellulose is then filtered and dried. A respective mass of 3, 6, 12, 15, 18, 25 and 40 g of calcium nitrate were dissolved for the preparation of the Ca-1 to Ca-7 samples, the sample Ca-8 was obtained with 40 g of calcium nitrate but with a shorter filtration time than Ca-7. The impregnated cellulose was pyrolyzed at 800 °C during 1 h under N_2_ with a heating ramp of 2 °C min^−1^ and a gas flow of 1 L min^−1^ in a vertical tubular oven (Carbolite tubular furnace). The resulting biochar was then collected and heated up to 1800 °C (1 h plateau) under N_2_ with a heating ramp of 2 °C min^−1^ and a gas flow rate of 300 L min^−1^ in a tubular furnace (Nabertherm RHTH 80/300/18). A commercial graphite (ChemPur CAS: 7782-42-5) was also studied as a reference of highly organized carbon.

The calcium concentration in the impregnated sample (before pyrolysis) was determined by Inductively Coupled Plasma Optical Emission Spectrometry (ICP-OES, Horiba Ultima 2) and defined as follows:

1 $$\%Ca\ (wt.\%)=\frac{{m}_{Ca}}{{m}_{Cellulose}+{m}_{Ca}}$$

The calcium concentrations of the samples are summarized in Table 1.

**Table 1:** Calcium concentration in the samples.

Sample	Non-impregnated	Ca-1	Ca-2	Ca-3	Ca-4	Ca-5	Ca-6	Ca-7	Ca-8
%Ca (wt.%)	< 0.01	0.32	0.95	1.37	2.12	2.83	3.14	3.83	4.39

### Study of the graphitization of biochars

#### X-ray diffraction

The macro and nanotexture of the graphenic structures in the samples was studied by X-Ray Diffraction (XRD, PANalytical X’pert Pro MPD) with a Cu-Kα radiation source (λ = 1.542 Å), operating at 45 kV and 40 mA. Diffraction peaks were recorded at 0.5°s^-1^ in the range 10°–100° in 2θ. The asymmetric *002* diffraction peak (2θ ≈ 24 °) of the biochars was fitted with two pseudo-Voigt functions. The symmetric *002* diffraction peak of graphite was fitted with a single pseudo-Voigt. The asymmetric *10* (2θ ≈ 43 °) and *11* (2θ ≈ 80 °) diffraction peaks were both fitted with a Breit–Wigner–Fano function. For the commercial graphite, the *100*, *101* and *110* diffraction peaks at 2θ ≈ 42.5 °, 2θ ≈ 44 ° and 2θ ≈ 78 ° respectively were fitted with a pseudo-Voigt function. The crystallite sizes of the graphenic structures L_a_ (in-plane length) and L_c_ (stacking height) were determined from the XRD spectra using the Scherrer equation^61^:

2 $$L \left(nm\right)=\frac{K\cdot \lambda }{\sqrt{{\beta }^{2}-{s}^{2}}\cdot cos\theta }$$

where *λ* is the radiation wavelength (0.1543 nm), *K* is a constant equal to 1.84 and 0.89 for L_a_ of the biochars and commercial graphite^62,63^ respectively. *K* is equal to 0.89 for the determination of L_c_ for all samples. *β* is the Full Width at Half Maximum (FWHM, in rad) of the *10* or *11* diffraction peaks for L_a_ and of the *002* peak for L_c_. *s* is the FWHM of a standard specimen (silica) to adjust for instrumental broadening. *θ* is the Bragg position of the *10* (*100* for graphite) and *002* diffraction peaks for L_a_ and L_c_ respectively. The mean value between L_a_ determined from the *10* and *11* peaks was taken. The interlayer spacing d_002_ was determined using Bragg’s law:

3 $${d}_{002}(nm)=\frac{\lambda }{2\cdot sin{\theta }_{002}}$$

The average number of stacked graphene fringes *n*_*fringes*_ in graphenic structures was obtained from L_c_ and d_002_ with:

4 $${n}_{fringes}=\frac{{L}_{c}}{{d}_{002}}+1$$

#### Raman spectroscopy

The structure and nanotexture of the samples were studied using Raman spectroscopy (*WITec Alpha 300R,* laser excitation = 532 nm). The analyses were performed with KBr/biochar pellets (weight ratio biochar/KBr = 0.025). Raman spectra were acquired by studying a 7 µm square (42 acquisitions/line, 42 lines/sample) on the surface of a sample particle. When relevant, the spectra acquisition was refined with the cluster option to obtain a spatial distribution of the various chemical signatures. The D and G bands (at 1350 cm^−1^ and 1680 cm^−1^ respectively) were both fitted with a Lorentzian function. The crystallites size L_a_ was estimated from the Raman results with the Tuinstra and Koenig formula^64^.

5 $${L}_{a} \left(nm\right)=\frac{4.4}{\frac{{I}_{D}}{{I}_{G}}}{\cdot \left(\frac{2.41}{{E}_{L}}\right)}^{\alpha }$$

where *E*_*L*_ is the laser energy (2.3308 eV) and *I* the intensity of the D and G bands. *α* is a unique coefficient for each sample to account for the laser dependency. The precise determination of α was not possible. As such, α was taken as 4 in this study, which correspond to the α value obtained for ordered graphite. Since the laser energy used in this study (2.3308 eV) is close to the energy used by Tuinstra and Koenig for their formula (2.41 eV), this will limit the influence of α on L_a_ estimation and will not impact the approximate size and variation tendency of L_a_.

#### High-resolution transmission electron microscopy

The structure, texture and nanotexture of the samples were examined at a nanoscopic scale using High-Resolution Transmission Electron Microscopy (HRTEM, *JEOL cold-FEG JEM-ARM200F*) operated at 200 kV equipped with a probe Cs corrector reaching a spatial resolution of 0.078 nm. HRTEM images were processed using a home-made image analysis numerical tool to obtain quantitative information about the graphene fringes. The algorithm of the tool was adapted from the method described by Yehliu et al.^65^. The mains steps of the algorithm are: *Selection of regions of interest*—Contrast improvement—*Image filtering using the Fourier transform—Binarization—Skeletonization—Correction of errors—Calculation of chosen parameters*. The tool was implemented in MATLAB software. The contrast of the entry image is first improved by black and white inversion and histogram equalization to better separate the fringes from the background. The image is then converted into the frequency domain with a fast Fourier transform and filtered with a gaussian lowpass filter. The cutoff frequency was selected as 3.333 nm^−1^ (0.300 nm)^−1^, meaning that objects separated by less than 0.300 nm are attenuated. As the interlayer spacing in a graphenic structure is usually greater than 0.3354 nm (d_002_ of hexagonal graphite), no fringes information is lost. A top-hat transformation is then applied to highlight high contrasted areas by correcting nonuniform illumination. The structural element for the top-hat transformation was a disk with an adjustable diameter larger than the apparent thickness of a single fringe to ensure the conservation of the fringes. The image is then binarized with a threshold determined using Otsu’s method and skeletonized with MATLAB built-in skeletonization algorithm. Finally, a home-made algorithm is applied to correct Y or X fringe ends and aligned fringes separated by less than 0.492 nm (two aromatic rings) are reconnected. The fringes shorter than 0.492 nm are too short to have a physical meaning and are removed.

The numerical tool measures the average crystallite size L_a_, the average fringes tortuosity (defined as the ratio of L_a_ on the shortest distance between the fringe extremities), the average interlayer spacing d_002_ and the number of graphene fringes stacked in a graphenic structure. With this numerical tool, a quantitative comparison of the HRTEM images analysis with the XRD and Raman spectroscopy results can be made.

## Results and discussion

### Investigation of the carbon crystalline organization

**Figure 1:**
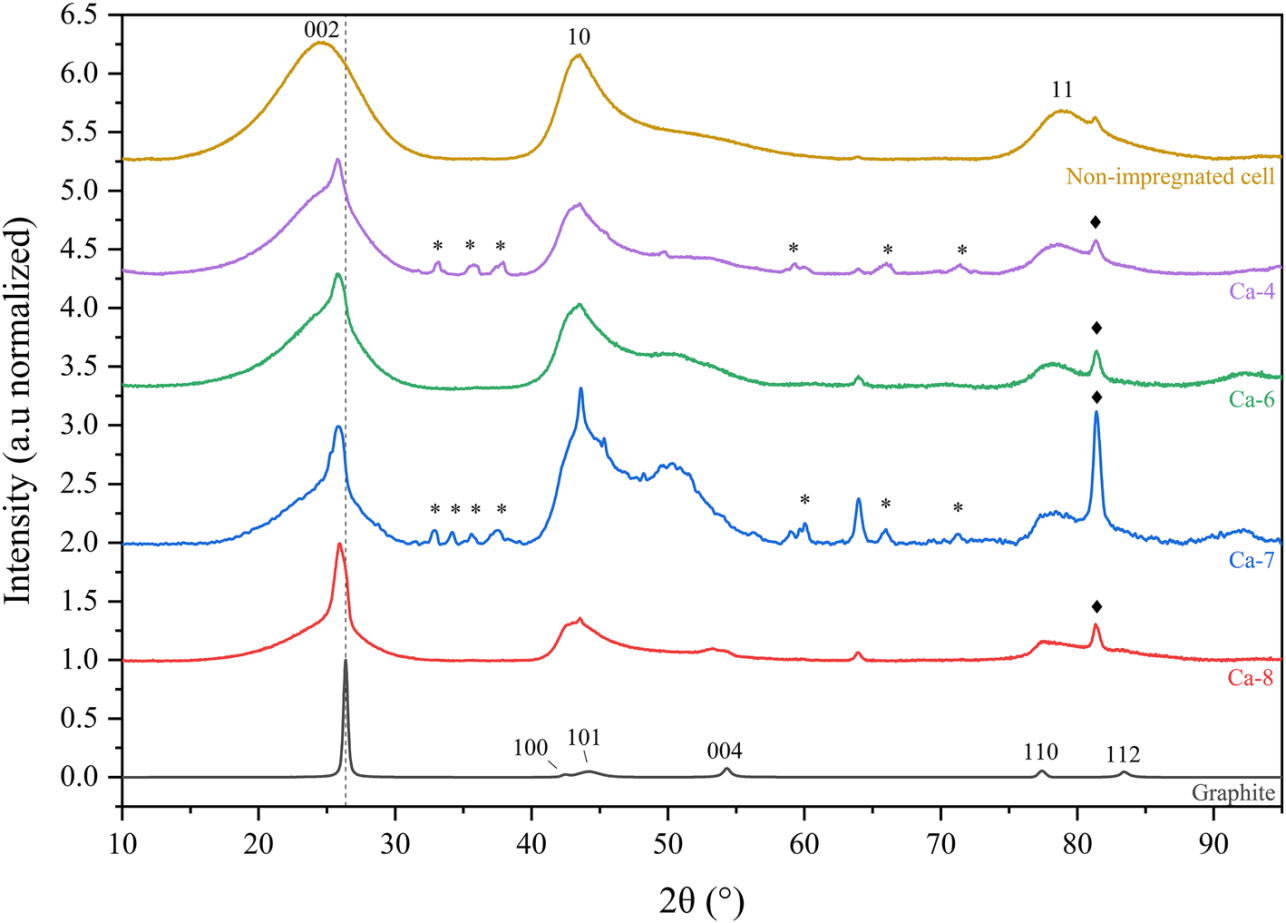
XRD patterns (λ = 1.542 Å) of non-impregnated, Ca-4, Ca-6, Ca-7, Ca-8 samples and commercial graphite. The peak label (asterisk) belongs to the Ca species. The peak label (closed diamond) belongs to the sample holder.

XRD provides insights on the average organization of the carbon matter in the samples. XRD patterns of the non-impregnated biochar and Ca-loaded biochars were acquired after graphitization and compared to a commercial graphite. Only the XRD patterns of the non-impregnated, Ca-4 (%Ca = 2.12 wt.%), Ca-7 (%Ca = 3.83 wt.%), Ca-8 (%Ca = 4.39 wt.%) and commercial graphite samples are presented in Fig. 1. The overall shape of the biochar patterns confirmed the production of carbonaceous turbostratic structures^66^ (structures with irregular stacking of the graphene fringes) and the presence of Ca mineral species (peaks label *) for the impregnated samples. Indeed, non-impregnated and impregnated samples exhibited a broad and asymmetric peak at the *002* and *10* positions while graphite exhibited a sharp and intense *002* peak and a clear distinction between the *101* and *110* peaks. The large broadness of the peaks indicated materials with small crystallite sizes (short-order organization of the graphenic structures) or high curvature of the graphene fringes, as XRD requires flatness of the crystalline structures. This results is known for hard carbon^66^. The *10* and *11* peaks of the non-impregnated and impregnated samples did not differ greatly. The *002* peak position and profile changed significantly with impregnation. The *002* peak shifted from 2θ = 24.65 ° for the non-impregnated biochar to 2θ = 25.99 ° for Ca-8. This shows a decrease in the interlayer spacing d_002_ to a value closer to the one for graphite (2θ = 26.37 °), which is taken as reference material. The tip of the *002* peak became sharper and a shoulder appeared on its left-side after impregnation. This asymmetry was previously reported for materials with heterogenous carbon structures^67–72^. The left-side of the *002* peak was attributed to poorly organized graphenic structures (randomly oriented texture and weak nanotexture) as found in hard carbons. The sharp tip of the *002* peak of the impregnated samples was attributed to highly organized graphenic structures (aligned texture, large nanotexture and graphene-like structure) as observed in graphitizable carbon or graphite.

**Figure 2:**
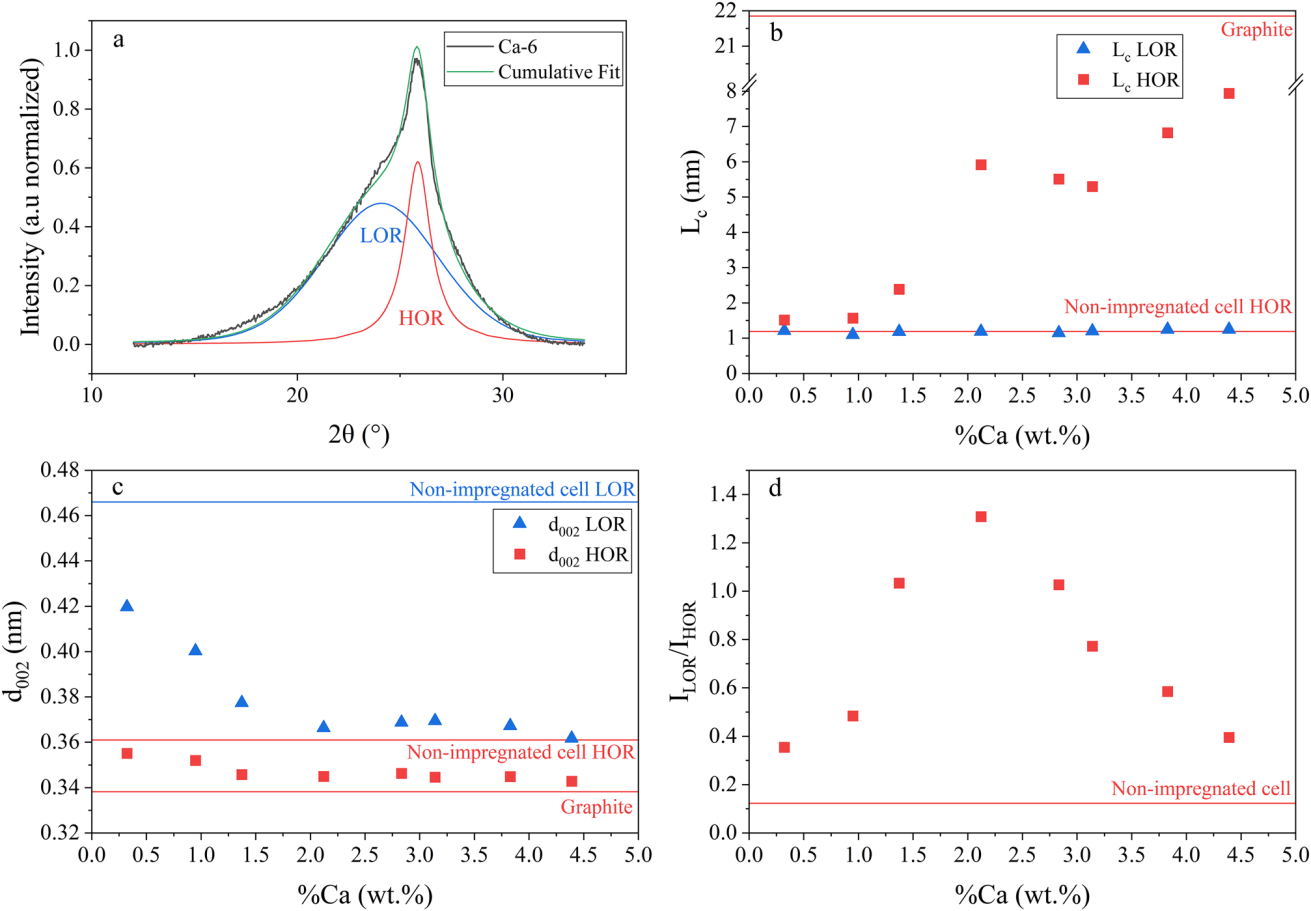
(**a**) Deconvolution of the XRD *002* peak of Ca-6 samples. (**b**) L_c_ of LOR and HOR as a function of %Ca. (**c**) d_002_ of LOR and HOR as a function of %Ca. (**d**) I_LOR_/I_HOR_ as a function of %Ca.

To highlight the different carbon structures in the biochars, the *002* peak was fitted with two pseudo-Voigt functions. The left-side referred to Lower-Organized Region (LOR) while the right-side indicated Higher-Organized Region (HOR) as shown in Fig. 2a. L_c_ (Eq. 2) and d_002_ (Eq. 3) were determined for both regions and are presented in Fig. 2b and c versus the initial calcium concentration.

LOR d_002_ decreased significantly, from 0.466 nm for the non-impregnated sample to a stable value around 0.369 nm for an initial calcium concentration superior to 2 wt.%. This value was slightly higher than the HOR d_002_ of the non-impregnated sample (d_002_ = 0.361 nm). HOR d_002_ showed the same profile, with an initial decrease to a stable value of 0.345 nm, close to that of the commercial graphite (d_002_ = 0.338 nm). Figure 2b shows LOR and HOR L_c_ versus Ca concentration. LOR L_c_ remained stable over the calcium concentration at the non-impregnated sample value (L_c_ = 1.24 nm). At the opposite, HOR L_c_ value increased for an initial calcium concentration below 2 wt.% and reached a plateau around 6 nm at an initial calcium concentration between 2 and 3 wt.%, then increased up to 8 nm at higher initial calcium concentrations. The average number of stacked graphene fringes (n_fringes_) increased from ≈ 4 fringes for the non-impregnated sample to ≈ 24 fringes for Ca-8 sample, meaning that calcium acted as a catalyst for cellulose graphitization in enhancing the graphenic structure rate.

Figure 2d shows the ratio between LOR and HOR peak intensities (I_LOR_/I_HOR_). It increased up to ≈ 2 wt.% of calcium and then decreased. This profile was unexpected since the HOR domain (and its peak intensity) would likely increase with Ca concentration, leading to a decrease of I_LOR_/I_HOR_. Indeed, highly organized graphenic structures developed with increasing calcium concentration, as it is confirmed by the high HOR L_c_ and low HOR d_002_ values for an initial calcium concentration superior to 2 wt.%. A possible explanation is a variation in the significance of the LOR and HOR peaks among the sample. For the non-impregnated sample, the *002* peak was mainly defined by the HOR peak (low I_LOR_/I_HOR_). The HOR peak corresponds to poorly organized graphenic structures (high d_002_ and small L_c_) as it was observed for hard carbon^73^, whereas the LOR peak was attributed to a small fraction of highly disorganized (amorphous) carbon structures^72^. With calcium impregnation, the disorganized carbon structures rate decreased in favor of much more organized graphene-like structures. For an initial calcium concentration below 2 wt.%, HOR peak mainly composed the *002* peak but the material exhibited a low L_c_ value (Fig. 2b) and a high d_002_ (Fig. 2c). In this stage, the HOR peak represented graphene-like structures with some poorly organized graphenic structures^71^. For initial calcium concentrations above 2 wt.%, the HOR peak represents exclusively graphene-like structures (low d_002_ and high L_c_) that mainly referred to regions of the biochar in contact with the calcium particles, while the LOR peak represents hard carbon structures, resulting from free or less contact regions with the catalyst during the heat treatment. In this stage, HOR d_002_ and L_c_ did not vary significantly but I_LOR_/I_HOR_ decreased. This could be attributed to a promoting effect of calcium in the conversion of LOR to HOR.

Therefore, additional calcium did not modify further the nanotexture of the graphenic structures but improved the rate of highly organized graphenic structures in the biochar.

Finally, the bulk structure was almost turbostratic and became more graphenic with calcium impregnation, which is confirmed by the quantified nanotextural parameters L_c_ (stacking), and d_002_. To better understand how the transformation occurred, we have also investigated the carbon organization at local scale.

### Investigation of the carbon organization at local scale

**Figure 3:**
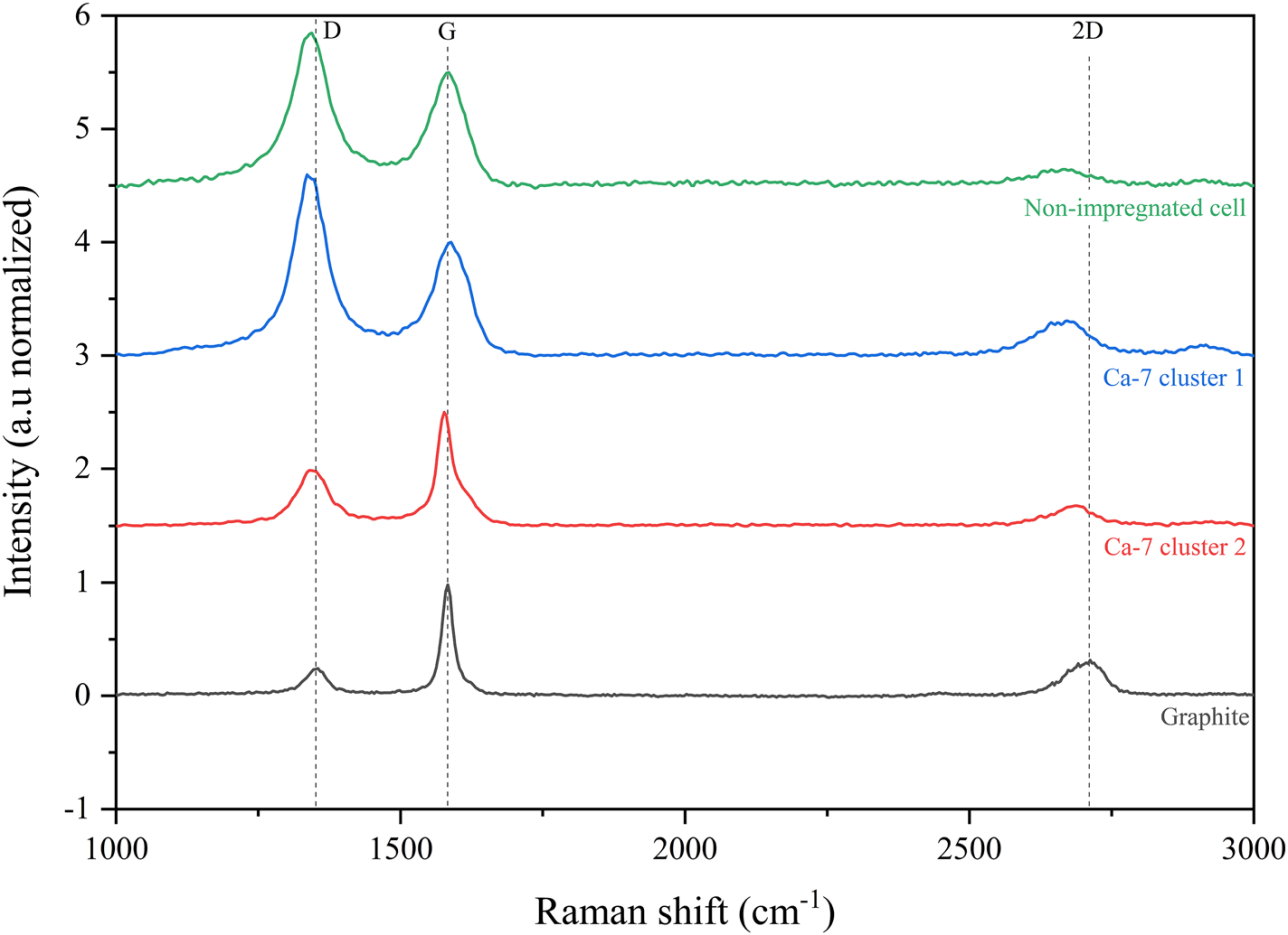
Raman spectra of non-impregnated cell, Ca-7 and commercial graphite samples.

At local scale, Raman spectroscopy gives chemical signature of the samples and can also highlight heterogeneity in chemical compositions. Only the non-impregnated cellulose biochar, Ca-7 and commercial graphite spectra are presented in Fig. 3. Raman spectra of Ca-4 and Ca-6 samples are provided in Supplementary material on Fig. S1. The spectrum of the non-impregnated cellulose biochar had a broad and intense D and G band (I_D_/I_G_ = 1.34) whereas the commercial graphite had a small D band and a narrow G band (I_D_/I_G_ = 0.23). G band refers to graphenic signature and the D band is likely attributed to defects in the graphene-like materials^74,75^. The defects include, with various intensities, edges, curvature or vacancies among others^74^. Graphite, which is composed of long-ordered graphenic arrangement has a weak D band compared to the G band. For all samples, the D and G bands were clearly separated, which indicated the absence of amorphous carbon through the valley between both bands^76,77^. For each impregnated samples, different chemical signatures, called clusters, could be identified. Two clusters of the Ca-7 sample are displayed in Fig. 3. Some clusters are close to the non-impregnated cellulose biochar spectrum (see Ca-7 cluster 1, I_D_/I_G_ = 1.40), this could correspond to spatial domains with limited effect of the catalyst. As these domains were highly defective, they could be attributed to the turbostratic structures previously evidenced by XRD. Some clusters showed a decrease and a thinning of the D band (see Ca-7 cluster 2, I_D_/I_G_ = 0.59), meaning a reduction of the defects in the graphene fringes and more organized graphenic structures. The second order bands were observed for all samples, due to the increased organization. The 2D band (double resonance of the D band) gives information on the texture and nanotexture of the graphenic structures using its shape, intensity and position^74^. The D band of the non-impregnated cellulose sample (1341 cm^−1^) was slightly red-shifted from the D band position of the commercial graphite (1351 cm^−1^). This shift may be due to defects or curvature of the graphene fringes^76^. The D band position of the impregnated samples ranged between the D band positions of the non-impregnated cellulose and commercial graphite samples, which implied a slightly less defective nanotexture. HRTEM images could give insights on the kind of defects (edges/curvature) involved.

**Figure 4:**
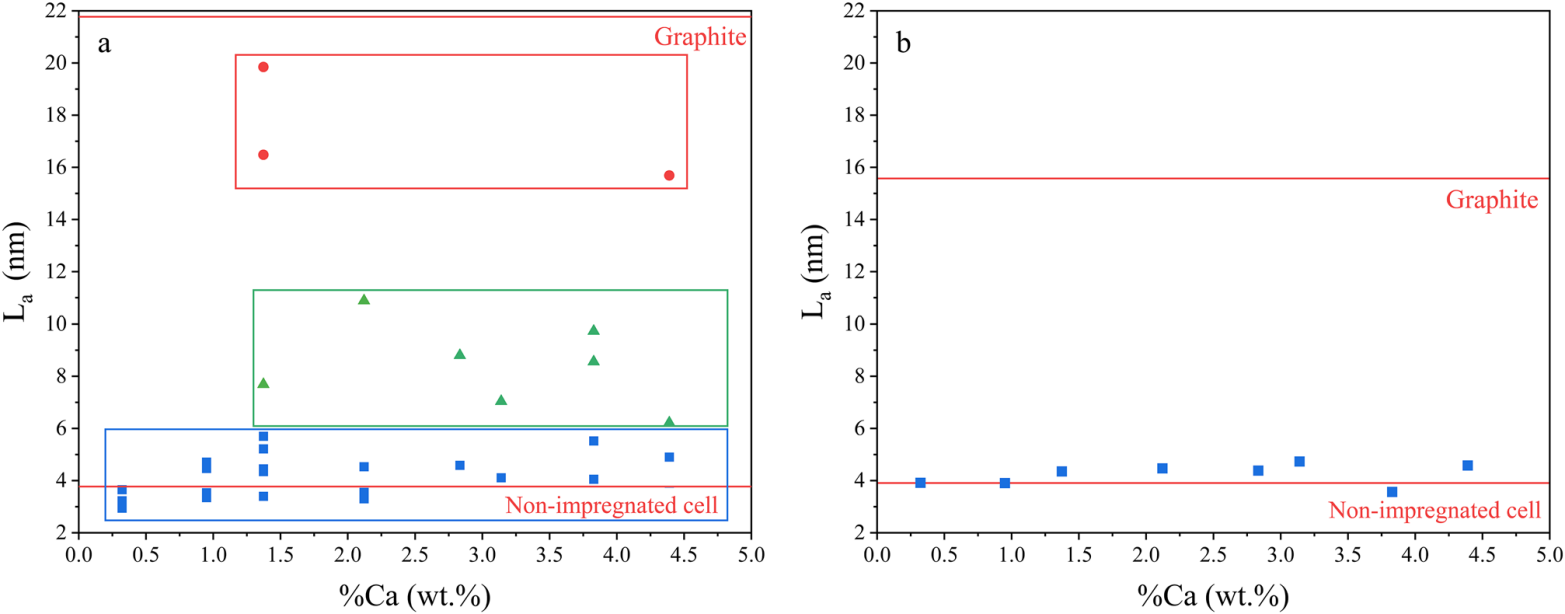
L_a_ as a function of %Ca from (**a**) Raman spectroscopy and (**b**) XRD analysis.

The crystallite size L_a_ (Eq. 5) was estimated for each cluster and the computed values are presented in Fig. 4a. L_a_ values were very heterogenous, however three kind of graphenic structures could be distinguished. For all samples, structures with L_a_ ranging between 3 and 6 nm were found (blue squares in Fig. 4a), close to the L_a_ determined for the non-impregnated sample (L_a_ = 3.77 nm). It could correspond to spatial domains with little or no catalyst, leading to graphenic structures with weak nanotexture, as observed in the non-impregnated sample. The second specific feature corresponds to larger graphenic structures, with L_a_ values around 9 nm (green triangles in Fig. 4a). The calcium impregnation led to the apparition of graphenic structures with a more developed nanotexture. Finally, structures with L_a_ values superior to 16 nm, very close to L_a_ of the commercial graphite were observed for the Ca-3 and Ca-8 samples (red circles in Fig. 4a), meaning that calcium can lead to the formation of highly organized graphenic structures. As these structures were not observed for the other samples, this level of organization is only marginal and can be explained by a local accumulation of calcium.

As Raman spectroscopy targets the carbon organization at the surface of the samples, no relation between L_a_ and %Ca could be established.

L_a_ values extracted from the XRD spectra (Eq. 2) are presented in Fig. 4b for comparison. These L_a_ values remained around the L_a_ value of the non-impregnated sample (3.91 nm). L_a_ values from XRD were in accordance with the smallest L_a_ values obtained from Raman analysis, which referred to poorly organized graphenic structures. Since two different graphenic structures (turbostratic and graphene-like) were present in the materials, the contribution to the observed *10* and *11* peaks of the turbostratic structures may have overshadowed those of the graphene-like structures, thus preventing a good estimation of L_a_ from XRD.

Raman spectroscopy and XRD both showed a heterogeneity of the carbon structures in the Ca-impregnated samples. XRD indicated the existence of graphenic structures composed of few units of graphene fringes (L_c_ < 2 nm) stacked with a turbostratic structures (d_002_ > 0.361 nm), similar to the non-impregnated sample. Raman spectroscopy completed this description by indicated a high density of defects (I_D_/I_G_ > 1) and poor fringes lengths (L_a_ < 6 nm) for this kind of graphenic domain. However, XRD informed about the existence of well-developed graphene-like structures (L_c_ ≈ 6 nm, d_002_ ≈ 0.345 nm). Raman spectroscopy described them as large (L_a_ > 6 nm) and low-defective (I_D_/I_G_ < 1).

Direct observation of the carbon materials at nanoscopic scale with HRTEM was expected to improve the understanding of the texture and nanotexture of the different carbon structures and complete the observations of the XRD and Raman spectroscopy. Especially, HRTEM should inform about the type of defects encountered in the graphenic structures.

### Nanoscale observation of the carbon organization

**Figure 5:**
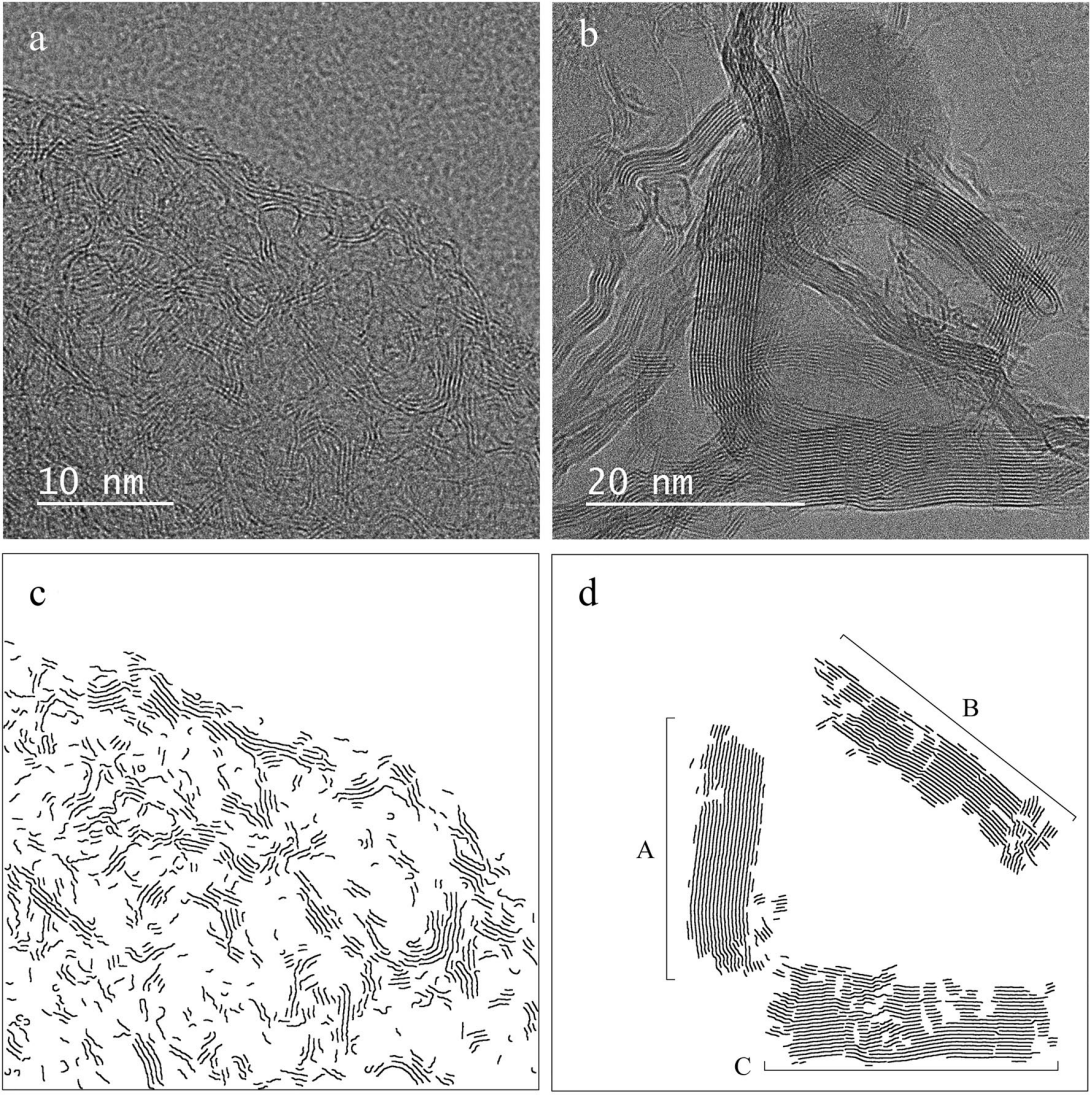
(**a**) HRTEM image of non-impregnated cellulose biochar (X500000). (**b**) HRTEM image of Ca-6 sample (X400000). (**c**) Processed image of non-impregnated cellulose biochar. (**d**) Processed image of Ca-6 sample image.

Images obtained from HRTEM enable the direct observation of the inner organization of the materials. HRTEM images were acquired for some samples (non-impregnated cellulose biochar, Ca-2, Ca-4, Ca-6 and Ca-7). Two of the most representative images, obtained from the non-impregnated cellulose biochar and Ca-6 sample, are presented in Fig. 5a and b. Additional images of Ca-4 and Ca-7 samples are available in Supplementary material on Fig. S2.

The image of the non-impregnated cellulose biochar (Fig. 5a) showed highly disorganized graphene fringes, grouped into small graphenic structures long of few nanometers with a poor stacking (< 4 graphene fringes). The graphene fringes were randomly oriented and highly curved, highlighting a weak structuration typical of hard carbons^78^. The strong curvature and short length (implying lot of edges) of the graphene fringes may explain the significant shift of the Raman D band of the non-impregnated cellulose biochar sample from the D band position of graphite, as well as the broad and asymmetric *10* and *11* bands in the XRD spectra. This specific graphene fringes organization was also observed for the impregnated samples.

For the impregnated samples, in addition to poorly organized structures, highly organized graphenic structures were observed (Fig. 5b), characterized by a high structuration and developed nanotexture with large and straight graphene fringes. The observed graphenic structures validated the previous XRD and Raman results and proved the apparition of graphene-like structures promoted by the calcium catalyst.

The disorganization of the graphenic structures in the non-impregnated cellulose biochar prevents a quantitative evaluation of the crystallite sizes of the graphenic structures from the analysis of the HRTEM images alone. That is why a home-made image analysis numerical tool was applied to the HRTEM images to extract some quantitative information about the graphenic structures. For Ca-impregnated samples images, the image analysis tool enables a precise description of each apparent graphene-like structures.

For the Ca-6 sample image, three distinguishable groups of graphene fringes were isolated prior running the tool, named A, B and C. The images obtained after application of the tool are presented in Fig. 5c and d.

Average L_a_ and d_002_ values, n_fringes_ and tortuosity value of the graphenic structures were estimated from the HRTEM images and are presented in Table 2. For Ca-6 sample, these values were estimated for each of the three domains and displayed separately in Table 2.

**Table 2:** Parameters of the graphenic structures from processing of the HRTEM images.

		L_a_ (nm)	d_002_ (nm)	n_fringes_	Tortuosity
Non-impregnated	HRTEM	1.39	0.370	3.3	1.18
Ca-6	HRTEM A	7.87	0.353	18	1.03
	HRTEM B	3.49	0.353	18	1.04
	HRTEM C	3.21	0.348	26	1.02

The interlayer spacing d_002_ computed from the HRTEM image of the non-impregnated cellulose biochar was equal to 0.370 nm, far above the d_002_ of graphite obtained from XRD (d_002_ = 0.338 nm) but close to the d_002_ of the hard carbon structures (d_002_ = 0.361 nm) determined from XRD. The average number of graphene fringes stacked n_fringes_ in the HRTEM image of the non-impregnated cellulose biochar was equal to 3.3 and the graphene fringes in this sample were also moderately curved (average tortuosity of 1.18). The carbon structures represented in the HRTEM image of the non-impregnated cellulose biochar show a good agreement with the results obtained by XRD and Raman spectroscopy for hard carbon, and highlights their poor organization, with random orientation and defective texture (short and curved fringes).

The three graphenic structures represented in the HRTEM image of the Ca-6 sample had a tortuosity below 1.04, an interlayer spacing d_002_ below 0.353 nm and a stacking up to 26 fringes. These values were in accordance with the crystallite sizes (d_002_ = 0.345 nm, n_fringes_ ≈ 21) computed from XRD for the graphene-like structures. The HRTEM image of Ca-6 sample confirmed the formation of well-organized graphenic structures with calcium impregnation.

L_a_ obtained from HRTEM images increased from 1.39 nm for the non-impregnated cellulose biochar to 3.21 nm (domain C) and 7.87 nm (domain A) for the Ca-6 sample image. Defects in domains B and C, such as superimpositions and crossings of the graphenic structures, led to fragmentation of the graphene fringes after image processing, explaining the shorter L_a_ values obtained for domains B and C compared to domain A. However, all the graphenic structures formed by calcium impregnation were composed of longer graphene fringes that for the non-impregnated cellulose biochar. The L_a_ calculated by the numerical tool was lower than the values of L_a_ previously reported from XRD and Raman spectroscopy. Only the length of the exposed side of the graphene fringes that are parallel to the direction of observation could be measured from the HRTEM images, whereas XRD and Raman spectroscopy measure the average length of the graphenic structures in the basal plane. As such, L_a_ calculated from the HRTEM images cannot be rigorously compared to L_a_ calculated from XRD and Raman spectroscopy.

The study of HRTEM images showed that calcium impregnation led to formation of long and straight graphene fringes, gathered in isotropic structures with multiple fringes stacked. All these observations confirmed the XRD and Raman spectroscopy analyses, that suggested the beneficial effect of calcium to form highly organized graphene-like structures at a relatively low temperature.

## Conclusion

Graphitization of cellulose, a poorly organized carbon structure, was achieved after impregnation with calcium, a non-conventional environmental and abundant catalyst. The graphitization was carried out at 1800 °C, below the standard temperature above 2000 °C for thermal synthesis of graphene-like materials. Outstanding investigation of the carbon structures of the samples considered was achieved by studying the biochar organization at macro, micro and nanoscale using X-ray diffraction, Raman spectroscopy and high-resolution transmission electron microscopy paired with an image analysis numerical tool. The results highlighted that calcium promotes the conversion of the hard carbon turbostratic structure into a well-organized and less-defective graphene-like structure.

The graphene-like structures had an in-plane length up to 20 nm and an interlayer spacing of 0.345 nm, near to the crystallite sizes of a standard graphite. An increase of the calcium concentration resulted in a slight improvement of the crystallite sizes of the graphene-like structures but drastically enhanced their rate in the biochar. Further research will focus on the description of the calcium–carbon interactions to better understand the catalytic graphitization mechanism for poorly organized carbon materials such as biomass for further applications.

## Supplementary Information

Supplementary Figures.

## Data Availability

The datasets generated during and/or analyzed during the current study are available from the corresponding author on reasonable request.
