# Supplementary material for: Calcium as an innovative and effective catalyst for the synthesis of graphene-like materials from cellulose
Source: Sci Rep. 2022 Dec 13;12:21492. doi: 10.1038/s41598-022-25943-3 (PMC9747789; doi:10.1038/s41598-022-25943-3)
Supplement: Supplementary file 1 — Supplementary Figures. [file 41598_2022_25943_MOESM1_ESM.pdf]

Calcium as an innovative and effective catalyst for the synthesis of graphene-like materials from cellulose

Théotime Béguerie[1], Elsa Weiss-Hortala[1], Ange Nzihou[1,2,3]*

[1] Université de Toulouse, Mines Albi, CNRS, Centre RAPSODEE, Campus Jarlard, Route de Teillet, F.81013 Albi Cedex 09, France

[2] Princeton University, School of Engineering and Applied Science, Princeton, NJ 08544, USA

[3] Princeton University, Andlinger Center for Energy and the Environment, Princeton, NJ 08544, USA

* ange.nzihou@mines-albi.fr, ange.nzihou@princeton.edu

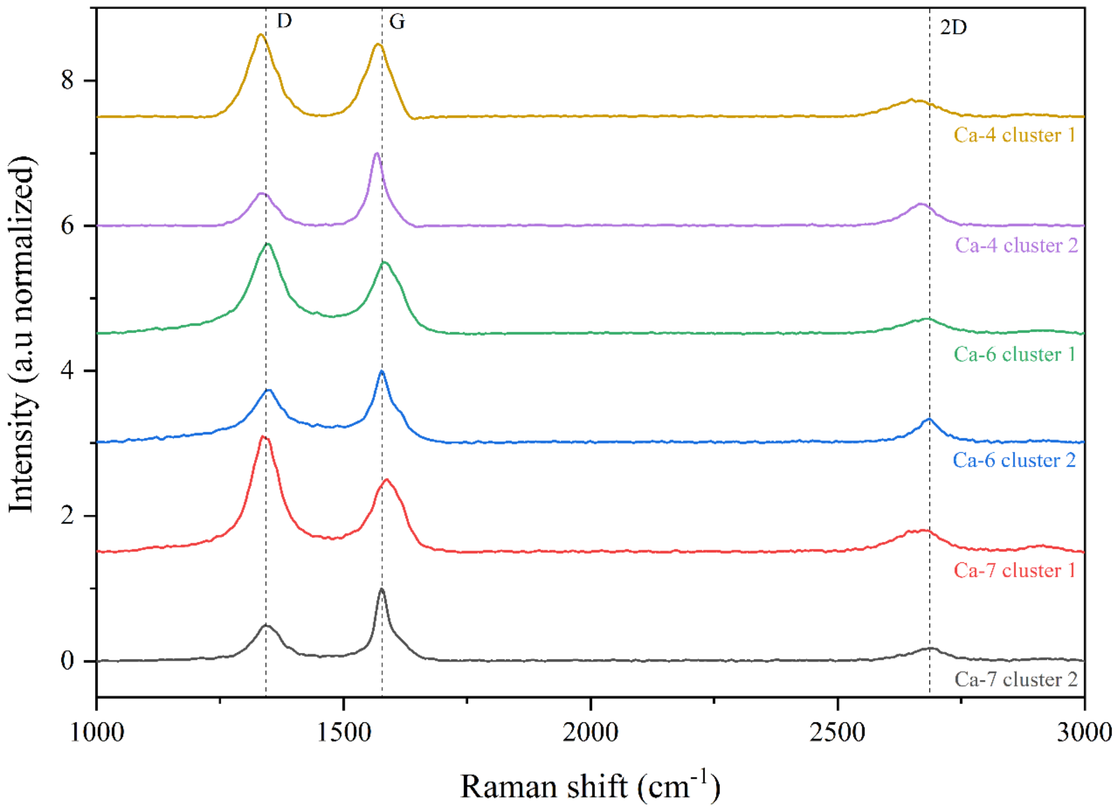

Supplementary Figure S1. Raman spectra of Ca-4, Ca-6 and Ca-7 samples.

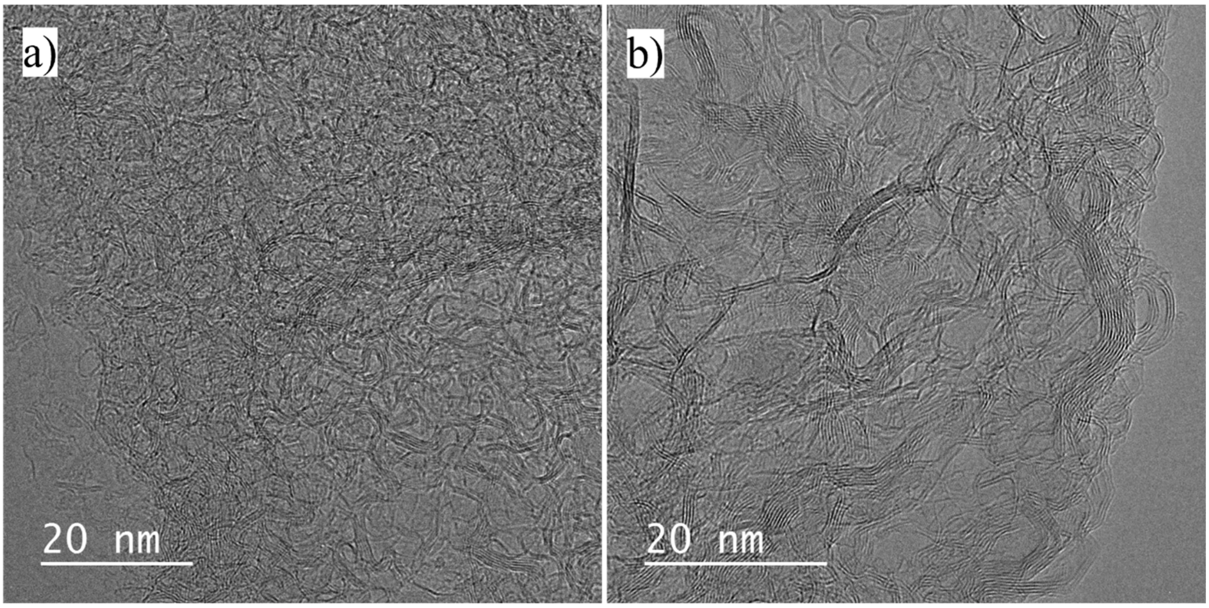

Supplementary Figure S2. a) HRTEM image of Ca-4 sample (X250000). **b**) HRTEM image of Ca-7 sample (X300000).
